# Supplementary figures and images for: Immigration and establishment of Trypanosoma cruzi in Arequipa, Peru
Source: PLoS One. 2019 Aug 27;14(8):e0221678. doi: 10.1371/journal.pone.0221678 (PMC6711515; doi:10.1371/journal.pone.0221678)

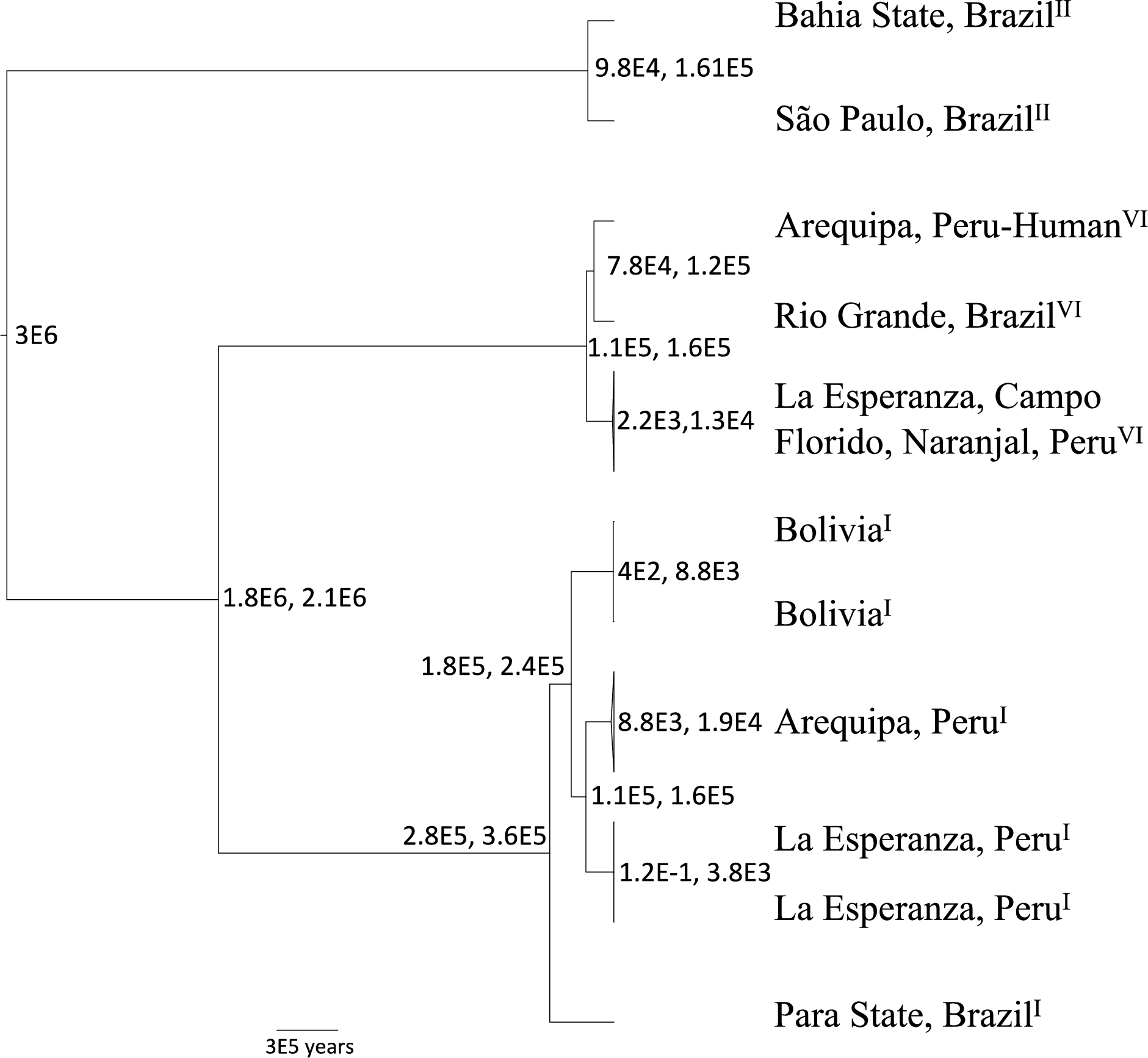

Supplement: S1 Fig — Estimates for divergence timing are displayed as 95% confidence intervals at each node. Estimates are based on rooting the phylogeny at 3mya. Sample collection locations are shown at the tips. The DTU each sample belongs to is labeled with its corresponding Roman Numeral in superscript. Due to the low diversity in maxicircle sequence among Arequipan isolates and the uncertainty in dating the tree root, care should be taken when interpreting divergence time estimates. (TIF) [file pone.0221678.s001.tif]

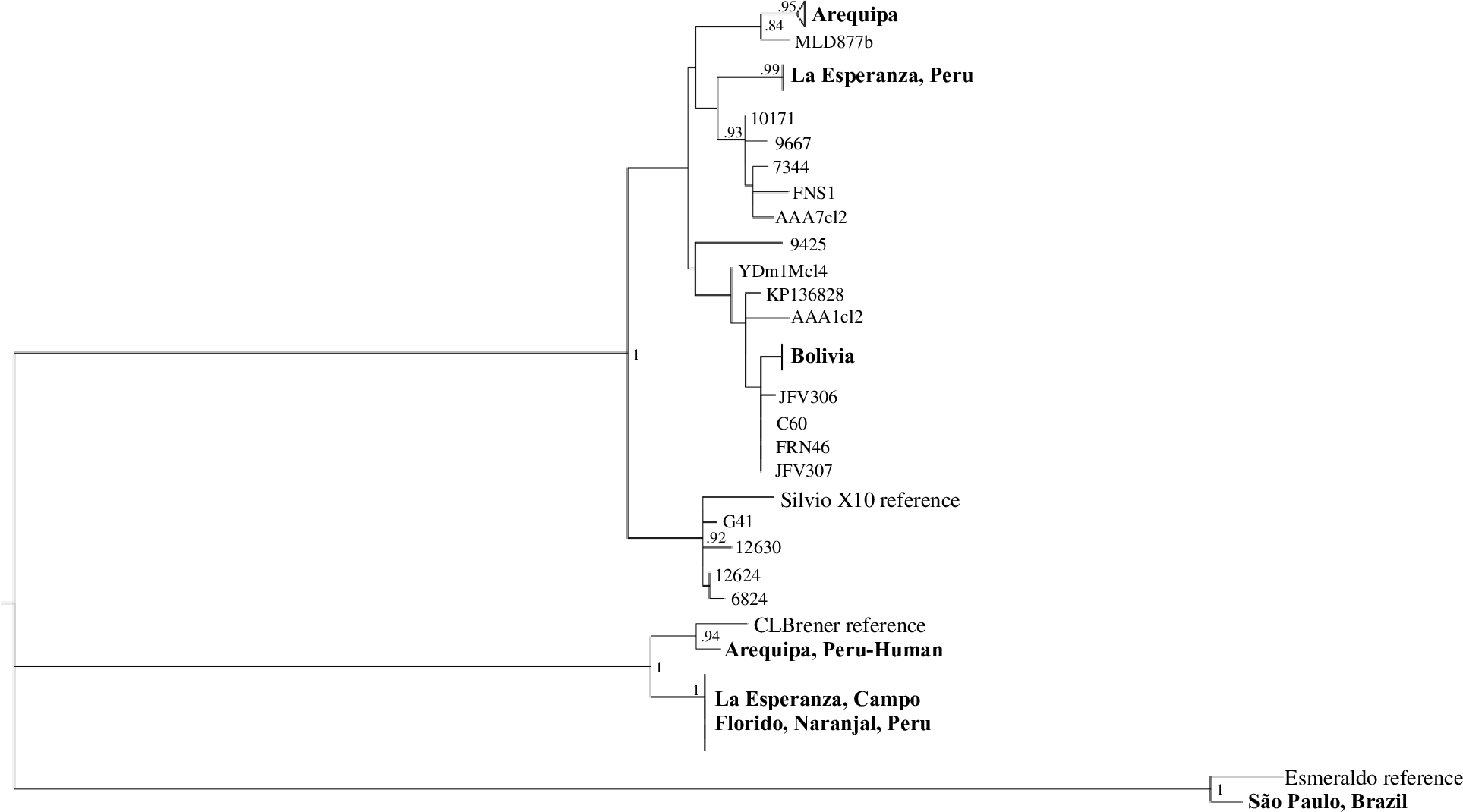

Supplement: S2 Fig — Short maxicircle sequences (<4.5kb) were not included in the analyses due to limited overlap with each other and the limited number of informative SNPs. The total alignment length was 2551 base pairs. Clades and branches that include samples sequenced here (Fig 1) are in bold font. Bootstrap support greater than 0.8 is labeled on nodes. (TIF) [file pone.0221678.s002.tif]

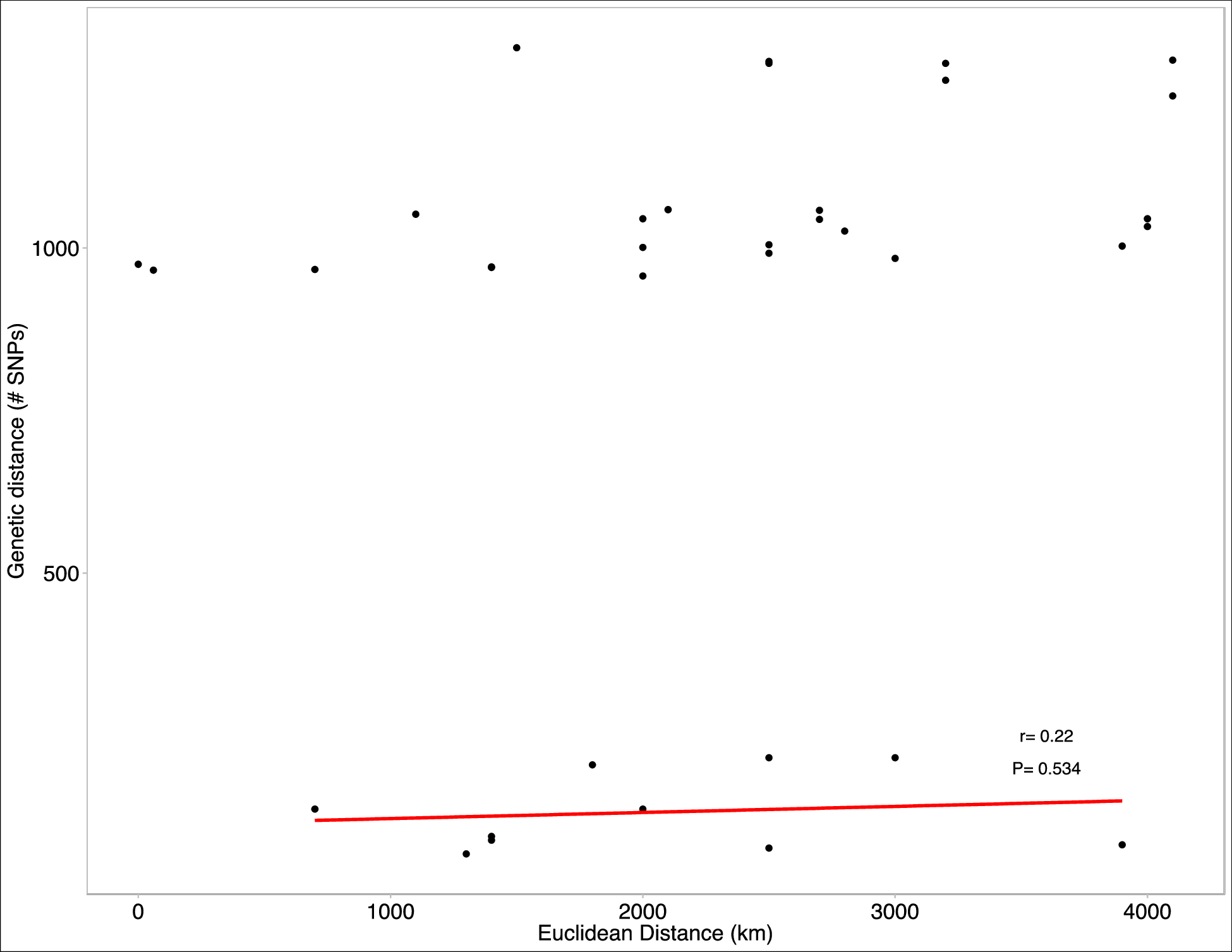

Supplement: S3 Fig — Only within-DTU distance data was used to perform the linear regression (red line). (TIF) [file pone.0221678.s003.tif]
