# Supplementary material for: Immigration and establishment of Trypanosoma cruzi in Arequipa, Peru
Source: PLoS One. 2019 Aug 27;14(8):e0221678. doi: 10.1371/journal.pone.0221678 (PMC6711515; doi:10.1371/journal.pone.0221678)
Supplement: S1 Table — (DOCX) [file pone.0221678.s004.docx]

**S1 Table. 18 additional partial maxicircle sequences obtained from NCBI**

| **Accession #** | **Label** |
| --- | --- |
| KM243352.1 | MLD877b |
| KM243354.1 | 10171 |
| KM243353.1 | 9667 |
| KM243356.1 | 7344 |
| KM243355.1 | FNS1 |
| JX123135.1 | AAA7cl2 |
| KM243351.1 | 9425 |
| JX123231.1 | YDm1Mcl4 |
| KP136828.1 | KP136828 |
| JX123145.1 | AAA1cl2 |
| KM243349.1 | JFV306 |
| KM243347.1 | C60 |
| KM243348.1 | FRN46 |
| KM243350.1 | JFV307 |
| KM243359.1 | G41 |
| KM243360.1 | 12630 |
| KM243357.1 | 12624 |
| KM243358.1 | 6824 |
