# Supplementary material for: Immigration and establishment of Trypanosoma cruzi in Arequipa, Peru
Source: PLoS One. 2019 Aug 27;14(8):e0221678. doi: 10.1371/journal.pone.0221678 (PMC6711515; doi:10.1371/journal.pone.0221678)
Supplement: S2 Table — (DOCX) [file pone.0221678.s005.docx]

**S2 Table. Sample Collection Data.**

| **Sample ID** | **House Number** | **District** | **Year Collected** | **Species Isolated From** | **SRA Accession No.** |
| --- | --- | --- | --- | --- | --- |
| TC126 | 1 | Bustamante | 2012 | *T. infestans* | SRS4304931 |
| TC125 | 2 | Bustamante | 2012 | *T. infestans* | SRS4304930 |
| TC050 | 3 | Bustamante | 2009 | Human | SRS4304912 |
| TC059 | 1 | Cajamarca | 2010 | *P. lignarius* | SRS4304892 |
| TC060 | 1 | Cajamarca | 2010 | *P. lignarius* | SRS4304893 |
| TC052 | 2 | Cajamarca | 2010 | *P. lignarius* | SRS4304899 |
| TC056 | 3 | Cajamarca | 2010 | *P. lignarius* | SRS4304895 |
| TC062 | 4 | Cajamarca | 2010 | Guinea Pig | SRS4304872 |
| TC054 | 5 | Cajamarca | 2010 | *P. lignarius* | SRS4304901 |
| TC122 | 1 | Cayma | 2011 | *T. infestans* | SRS4304927 |
| TC119 | 2 | Cayma | 2011 | *T. infestans* | SRS4304925 |
| TC135 | NA | Huanca | 2013 | *T. infestans* | SRS4304983 |
| TC143 | NA | Huanca | 2015 | *T. infestans* | SRS4304885 |
| TC149 | NA | Huanca | 2015 | *T. infestans* | SRS4304906 |
| TC142 | NA | Huanca | 2015 | *T. infestans* | SRS4304884 |
| TC141 | NA | Huanca | 2015 | *T. infestans* | SRS4304887 |
| TC148 | NA | Huanca | 2015 | *T. infestans* | SRS4304907 |
| TC145 | NA | Huanca | 2015 | *T. infestans* | SRS4304883 |
| TC151 | NA | Huanca | 2015 | *T. infestans* | SRS4304908 |
| TC150 | NA | Huanca | 2015 | *T. infestans* | SRS4304909 |
| TC152 | NA | Huanca | 2015 | *T. infestans* | SRS4304903 |
| TC144 | NA | Huanca | 2015 | *T. infestans* | SRS4304882 |
| TC146 | NA | Huanca | 2015 | *T. infestans* | SRS4304890 |
| TC015 | 1 | La Joya | 2008 | *T. infestans* | SRS4304941 |
| TC033 | 2 | La Joya | 2008 | *T. infestans* | SRS4304960 |
| TC029 | 3 | La Joya | 2008 | *T. infestans* | SRS4304952 |
| TC004 | 3 | La Joya | 2008 | *T. infestans* | SRS4304938 |
| TC037 | 4 | La Joya | 2008 | *T. infestans* | SRS4304945 |
| TC022 | 5 | La Joya | 2008 | *T. infestans* | SRS4304956 |
| TC019 | 6 | La Joya | 2008 | *T. infestans* | SRS4304959 |
| TC027 | 7 | La Joya | 2008 | *T. infestans* | SRS4304953 |
| TC034 | 8 | La Joya | 2008 | *T. infestans* | SRS4304946 |
| TC038 | 9 | La Joya | 2008 | *T. infestans* | SRS4304950 |
| TC031 | 10 | La Joya | 2008 | *T. infestans* | SRS4304961 |
| TC023 | 11 | La Joya | 2008 | Guinea Pig | SRS4304955 |
| TC035 | 12 | La Joya | 2008 | *T. infestans* | SRS4304947 |
| TC036 | 13 | La Joya | 2008 | *T. infestans* | SRS4304944 |
| TC014 | 14 | La Joya | 2008 | *T. infestans* | SRS4304936 |
| TC039 | 15 | La Joya | 2008 | *T. infestans* | SRS4304951 |
| TC001 | 16 | La Joya | 2008 | *T. infestans* | SRS4304935 |
| TC020 | 16 | La Joya | 2008 | *T. infestans* | SRS4304958 |
| TC002 | 16 | La Joya | 2008 | *T. infestans* | SRS4304934 |
| TC016 | 17 | La Joya | 2008 | *T. infestans* | SRS4304940 |
| TC026 | 18 | La Joya | 2008 | *T. infestans* | SRS4304954 |
| TC003 | 19 | La Joya | 2008 | *T. infestans* | SRS4304939 |
| TC040 | 20 | La Joya | 2008 | *T. infestans* | SRS4304948 |
| TC129 | 20 | La Joya | 2013 | *T. infestans* | SRS4304989 |
| TC130 | 20 | La Joya | 2013 | *T. infestans* | SRS4304988 |
| TC131 | 20 | La Joya | 2013 | *T. infestans* | SRS4304987 |
| TC147 | 20 | La Joya | 2015 | *T. infestans* | SRS4304891 |
| TC155 | 20 | La Joya | 2015 | *T. infestans* | SRS4304904 |
| TC088 | 1 | Mariano Melgar | 2011 | *T. infestans* | SRS4304859 |
| TC107 | 2 | Mariano Melgar | 2011 | *T. infestans* | SRS4304967 |
| TC099 | 3 | Mariano Melgar | 2011 | *T. infestans* | SRS4304980 |
| TC090 | 4 | Mariano Melgar | 2011 | *T. infestans* | SRS4304972 |
| TC097 | 5 | Mariano Melgar | 2011 | *T. infestans* | SRS4304976 |
| TC044 | 6 | Mariano Melgar | 2010 | Dog | SRS4304916 |
| TC055 | 6 | Mariano Melgar | 2010 | Guinea Pig | SRS4304894 |
| TC102 | 6 | Mariano Melgar | 2011 | *T. infestans* | SRS4304979 |
| TC098 | 7 | Mariano Melgar | 2011 | *T. infestans* | SRS4304977 |
| TC100 | 8 | Mariano Melgar | 2011 | *T. infestans* | SRS4304861 |
| TC110 | 9 | Mariano Melgar | 2011 | *T. infestans* | SRS4304965 |
| TC108 | 10 | Mariano Melgar | 2011 | *T. infestans* | SRS4304966 |
| TC101 | 11 | Mariano Melgar | 2011 | *T. infestans* | SRS4304978 |
| TC115 | 12 | Mariano Melgar | 2011 | *T. infestans* | SRS4304923 |
| TC071 | 13 | Mariano Melgar | 2011 | *T. infestans* | SRS4304866 |
| TC074 | 14 | Mariano Melgar | 2011 | *T. infestans* | SRS4304865 |
| TC072 | 15 | Mariano Melgar | 2011 | *T. infestans* | SRS4304867 |
| TC105 | 16 | Mariano Melgar | 2011 | *T. infestans* | SRS4304969 |
| TC064 | 17 | Mariano Melgar | 2010 | Guinea Pig | SRS4304878 |
| TC010 | 18 | Mariano Melgar | 2008 | *T. infestans* | SRS4304937 |
| TC111 | 19 | Mariano Melgar | 2011 | *T. infestans* | SRS4304964 |
| TC049 | 20 | Mariano Melgar | 2010 | *T. infestans* | SRS4304913 |
| TC076 | 20 | Mariano Melgar | 2011 | *T. infestans* | SRS4304863 |
| TC077 | 20 | Mariano Melgar | 2011 | *T. infestans* | SRS4304870 |
| TC078 | 20 | Mariano Melgar | 2011 | *T. infestans* | SRS4304871 |
| TC041 | 21 | Mariano Melgar | 2010 | Dog | SRS4304942 |
| TC116 | 22 | Mariano Melgar | 2011 | *T. infestans* | SRS4304924 |
| TC080 | 23 | Mariano Melgar | 2011 | *T. infestans* | SRS4304853 |
| TC104 | 23 | Mariano Melgar | 2011 | *T. infestans* | SRS4304970 |
| TC112 | 23 | Mariano Melgar | 2011 | *T. infestans* | SRS4304963 |
| TC073 | 24 | Mariano Melgar | 2011 | *T. infestans* | SRS4304864 |
| TC042 | 25 | Mariano Melgar | 2010 | Guinea Pig | SRS4304943 |
| TC043 | 25 | Mariano Melgar | 2010 | Guinea Pig | SRS4304914 |
| TC045 | 25 | Mariano Melgar | 2010 | Guinea Pig | SRS4304919 |
| TC061 | 25 | Mariano Melgar | 2010 | Guinea Pig | SRS4304874 |
| TC075 | 25 | Mariano Melgar | 2011 | *T. infestans* | SRS4304862 |
| TC070 | 25 | Mariano Melgar | 2011 | *T. infestans* | SRS4304869 |
| TC079 | 26 | Mariano Melgar | 2011 | *T. infestans* | SRS4304854 |
| TC068 | 27 | Mariano Melgar | 2011 | *T. infestans* | SRS4304881 |
| TC069 | 27 | Mariano Melgar | 2011 | *T. infestans* | SRS4304868 |
| TC095 | 28 | Mariano Melgar | 2011 | *T. infestans* | SRS4304975 |
| TC048 | 29 | Mariano Melgar | 2010 | *T. infestans* | SRS4304920 |
| TC081 | 30 | Mariano Melgar | 2011 | *T. infestans* | SRS4304856 |
| TC082 | 30 | Mariano Melgar | 2011 | *T. infestans* | SRS4304855 |
| TC085 | 30 | Mariano Melgar | 2011 | *T. infestans* | SRS4304852 |
| TC089 | 30 | Mariano Melgar | 2011 | *T. infestans* | SRS4304858 |
| TC084 | 30 | Mariano Melgar | 2011 | *T. infestans* | SRS4304849 |
| TC086 | 30 | Mariano Melgar | 2011 | *T. infestans* | SRS4304851 |
| TC103 | 30 | Mariano Melgar | 2011 | *T. infestans* | SRS4304971 |
| TC047 | 31 | Mariano Melgar | 2010 | *T. infestans* | SRS4304921 |
| TC083 | 31 | Mariano Melgar | 2011 | *T. infestans* | SRS4304850 |
| TC124 | 31 | Mariano Melgar | 2012 | *T. infestans* | SRS4304929 |
| TC065 | 32 | Mariano Melgar | 2011 | *T. infestans* | SRS4304860 |
| TC091 | 32 | Mariano Melgar | 2011 | *T. infestans* | SRS4304973 |
| TC092 | 32 | Mariano Melgar | 2011 | *T. infestans* | SRS4304974 |
| TC046 | 33 | Mariano Melgar | 2010 | *T. infestans* | SRS4304918 |
| TC132 | 1 | Miraflores | 2013 | *T. infestans* | SRS4304986 |
| TC051 | 2 | Miraflores | 2010 | *T. infestans* | SRS4304898 |
| TC134 | 3 | Miraflores | 2013 | *T. infestans* | SRS4304984 |
| TC133 | 4 | Miraflores | 2013 | *T. infestans* | SRS4304985 |
| TC137 | 5 | Miraflores | 2014 | *T. infestans* | SRS4304981 |
| TC136 | 6 | Miraflores | 2014 | *T. infestans* | SRS4304982 |
| TC113 | 1 | Sachaca | 2011 | *T. infestans* | SRS4304962 |
| TC114 | 1 | Sachaca | 2011 | *T. infestans* | SRS4304922 |
| TC123 | 2 | Sachaca | 2012 | *T. infestans* | SRS4304928 |
| TC067 | 3 | Sachaca | 2011 | *T. infestans* | SRS4304857 |
| TC106 | 3 | Sachaca | 2011 | *T. infestans* | SRS4304968 |
| TC127 | 1 | Tiabaya | 2012 | *T. infestans* | SRS4304990 |
| TC139 | 2 | Tiabaya | 2015 | *T. infestans* | SRS4304889 |
| TC154 | 3 | Tiabaya | 2015 | *T. infestans* | SRS4304905 |
| TC156 | 3 | Tiabaya | 2015 | *T. infestans* | SRS4304911 |
| TC153 | 4 | Tiabaya | 2015 | *T. infestans* | SRS4304902 |
| TC053 | 5 | Tiabaya | 2010 | *T. infestans* | SRS4304900 |
| TC058 | 6 | Tiabaya | 2010 | *T. infestans* | SRS4304897 |
| TC057 | 7 | Tiabaya | 2010 | *T. infestans* | SRS4304896 |
| TC140 | 8 | Tiabaya | 2015 | *T. infestans* | SRS4304886 |
| TC063 | 1 | Uchumayo | 2010 | *T. infestans* | SRS4304879 |
| TC120 | 1 | Uchumayo | 2011 | *T. infestans* | SRS4304926 |
| TC138 | 1 | Vitor | 2013 | *T. infestans* | SRS4304888 |
| Bol-DH29 | 1 | Bolivia | NA | Human | SRS4304910 |
| Bol-SH001 | 2 | Bolivia | NA | Human | SRS4304876 |
| TC-y | 1 | Sao Paulo, Brazil | 1950 | Human | SRS4304877 |
